# Synergetic static and radiation shielding characteristics of non-conventional concrete mixtures

**DOI:** 10.1038/s41598-025-28121-3

**Published:** 2025-12-02

**Authors:** Hazem K. A. Amin, Moamen G. El-Samrah, Nabil H. Amer, A. F. Tawfic, Mohamed A. E. M. Ali

**Affiliations:** 1 Civil Engineering Department, Military Technical College, Cairo, 11765 Egypt; 2 Nuclear Engineering Department, Military Technical College, Cairo, 11765 Egypt

**Keywords:** Concrete, Gamma-radiation, Fast neutron, Shielding, Engineering, Materials science, Physics

## Abstract

Concrete is extensively utilized in various applications related to radiation shielding, including nuclear power facilities, radiation shelters, high-voltage radiotherapy rooms, and for the transportation and storage of radioactive waste. Thus, this study developed eleven different concrete mixtures to evaluate their applicability for such critical applications. The control concrete mixture (NC) was made with typical concrete ingredients. Then, a (GC) specimen was made by fully substituting conventional dolomite with goethite aggregate. Thereafter, various concrete mixtures (GL, GR, and GT) were developed to determine the optimal replacement percentage of silica sand with leucoxene, rutile, and tourmaline powders, at replacement percentages of 10%, 20%, and 30%. The optimized results were drawn based on the mechanical and radiation shielding properties of concrete. The results indicated that the goethite concrete mixture exhibited acceptable compressive strength with negligible reduction relative to that of normal concrete. At the same time, all fine powders’ concrete demonstrated a general decrease in compressive strength by up to 41.95%. The incorporation of the materials. Radiation-shielding properties were assessed through experimental and computational analyses. The effective atomic number (Z_eff_) and linear attenuation coefficient (µ) were determined using Auto-Zeff and EpiXS software, respectively, across the photon energy range of 10 keV to 10 MeV. Goethite-based concretes, particularly GL30 and GR30, exhibited enhanced γ-ray attenuation due to their higher content of Fe and Ti and elevated densities. Fast neutron and total gamma-ray attenuation were experimentally measured using a Stilbene scintillation detection system. The GL30 and GT30 mixes demonstrated the highest fast neutron removal cross-sections (Σ_R_), supported by their superior densities and Fe/Ti contents. Furthermore, tourmaline-based mixes (GT20 and GT30) exhibited the best total γ-ray attenuation, attributed to the boron content in tourmaline, which effectively reduces secondary gamma emissions. Thermal neutron absorption tests, supported by JANIS-4.1 calculations, revealed that GT mixes achieved exceptional macroscopic absorption cross-sections, up to 2923% higher than normal concrete, owing to the ¹⁰B (n,α)⁷Li reaction. Overall, incorporating goethite with leucoxene or tourmaline powders up to 30% yields concretes suitable for both structural and radiation-shielding applications.

## Introduction

Radiation is a significant concern in structural and human safety. It includes ionized and non-ionized radiations. The latter radiation encompasses electromagnetic waves with a wavelength of 10 nm or greater, whereas ionizing radiation comprises X-rays, γ-rays, and subatomic particles^1^. Ionizing radiation can be classified into direct and indirect ionizing radiation. The primary concern with ionizing radiations, particularly γ-rays and neutrons, is their powerful and extended ranges, which have harmful effects on humans and structures. High-density materials, such as steel, bismuth, and lead, are recommended for attenuating gamma radiation due to their high density^2^. For neutron shielding, high-hydrogen-content materials can be combined with high-Z elements and thermal neutron absorbers, known as neutron poisons^2^.

The ideal shielding barrier should have a high density and concentration of hydrogen. Concrete shields, particularly those made of heavyweight concrete, are the most suitable contender due to their balance between density and hydrogen content. This type of concrete is commonly used as a shielding barrier in nuclear power plants, shelters, and radiotherapy megavoltage rooms, as well as for transporting radioactive waste. The selection of appropriate local aggregates and additives is crucial for achieving the desired mechanical and radiation attenuation characteristics^3^.

Over the past few decades, extensive research has been conducted using various concrete constituents to develop shielding barriers with high physical and mechanical properties, providing high levels of attenuation and protection against gamma rays and neutrons. For instance, the efficacy of nuclear radiation shielding barriers using different plaster constituents (Titanium, barite, steel slag, and hematite powders) was investigated by M.A.E.M. Ali et al. (2025)^4^. Results revealed the applicability of steel slag-titanium plaster due to its superior radiation-shielding properties. It also meets the requirements of a sustainable design. Moreover, T. Daungwilailuk et al. (2022)^5–8^ studied the protection of concrete against gamma and fast-neutron radiation, finding that barite enhances shielding capabilities and specimen strength, while cut steel bars significantly improve its static performance. In addition, B. Pomaro et al. (2019)^9^ studied the radiation shielding capabilities of two types of heavyweight concrete: one that contains barite and another that contains Electric Arc Furnace (EAF) slag. Compared to other studied combinations, EAF concrete achieved gamma rays’ attenuation better than that of barite concrete. Likewise, T. Shams et al. (2018)^10^ investigated gamma ray shields made of aggregates of barite and hematite of varying grades. The addition of non-conventional aggregates enhances both the linear attenuation coefficient and the compressive strength after 28 days. Also, they found that the layer order is insignificant. Compared to the multilayer shield, the one made of integrated mixed concrete attenuated radiation better.

Additionally, M. A. Abdelgawad et al. (2023)^11^ investigated the ability of thick concrete mixes, including barite and tourmaline, to attenuate nuclear radiation. Various proportions of tourmaline ore were used in the mixtures, ranging from 0% to 50%. The mechanical characteristics and neutron attenuation capabilities of the concrete were found to be substantially enhanced upon the addition of tourmaline ore. Additionally, I. M. Nabil et al. (2023)^12–15^ evaluated three concrete mixes with different aggregate types (dolomite, barite, and limonite) and boron carbide additives for radiation shielding. Results showed that barite mixes outperform dolomite in photon attenuation, while barite with an additive is superior in shielding against photons, fast neutrons, thermal neutrons, and secondary emitted γ-rays. Besides that, C. Thomas et al. (2019)^16^ examined the application of specialized concrete walls for constructing protective barriers against nuclear radiation. The concrete, containing natural limestone aggregate, polyvinyl alcohol fibers, and boron carbide, has demonstrated mechanical features, durability, and a suitable response to rapid temperature rises, making it ideal for the intended use. Additionally, the shielding properties of high-density concrete with ferro-boron as a substitute for granite aggregate were evaluated by M. K. A. Roslan et al. (2019)^17^. Results showed reduced workability and increased density, likely accompanied by improved strength and radiation shielding. Additionally, M. G. El-Samrah et al. (2022)^18–21^ investigated the gamma ray attenuation capability of various concrete mixtures using dolomite, barite, ilmenite, and celestite aggregates, along with two cementing materials. They found that the mixtures comprising barite and celestite were the most efficacious in attenuating gamma rays. In addition, M.A. Masoud et al. (2020)^22^ combined serpentine aggregate with hematite/barite aggregate at 25% and 50% ratios. The findings indicated that the use of boron and hydrogen harmed the physical characteristics while enhancing shielding characteristics.

Furthermore, J. Han et al. (2022)^23–27^ conducted a study on magnetite ultra-high-performance concrete using magnetite fine aggregate as a replacement for river sand at different replacement ratios. Experimental findings demonstrate enhanced work efficiency and superior gamma ray shielding capabilities. Similarly, K. Gunoglu and İ. Akkurt (2021)^28^ assessed linear attenuation coefficients of concrete using varying proportions of basalt-magnetite aggregates across different gamma energy levels. The results showed a linear correlation between the linear attenuation coefficients and the basalt-magnetite ratio in concrete, as well as the efficacy of radiation protection. In addition, M. Falahatkar Gashti et al. (2023)^29^ examined the radiation shielding characteristics of green concrete with magnetite aggregates in both standalone and cover-layer configurations. Results indicate that substituting conventional aggregates with magnetite and using steel fibers enhanced the linear attenuation coefficient. Additionally, M. A. E. M. Ali et al. (2023)^30^ investigated the mechanical characteristics and radiation attenuation performance of concrete mixes with ilmenite or tourmaline aggregates. Results indicate that ilmenite concrete generally decreases the strength of concrete, but tourmaline exhibits enhanced strength. Also, the samples exhibit significant attenuation of gamma and neutron emissions. Similarly, Egyptian black sand mineral products (zircon, black sand concentrate, magnetite, green silica, and ilmenite grade 2) were examined by T. G. Mohamed et al. (2024)^31^ for their potential application in the formulation of radiation shielding building materials. These products were deemed suitable, with magnetite and ilmenite identified as the most appropriate options. T. G. Mohamed et al. (2023)^32^ investigated the use of Black Sand Company supplies (magnetite and ilmenite) in concrete mixtures to create self-consolidating concrete (SCC). Eight different mixtures were tested for mechanical and physical properties, and compressive strength was evaluated at various temperatures. Finite element analysis (FEA) revealed that magnetite significantly enhances the strength and homogeneity of concrete mix design, and a temperature increase does not substantially affect the compressive strength. Additionally, A. M. Zeyad et al. (2022)^33^ investigated the characteristics of ultra-high-performance and heavyweight radiation shielding concrete using different aggregates and fibers. Lead fiber and magnetite aggregates exhibited superior radiation shielding characteristics.

On the other hand, the use of by-product materials in sustainable concrete production has revealed superior static and dynamic performance, as well as radiation attenuation capabilities, by replacing crushed stone aggregates with steel slag and silica sand. Thus, it was carried out by A. M. Amin, M. A. E. M. Ali, et al. (2023)^34^. Likewise, K. G. Mahmoud et al. (2023)^35^ produced four concrete specimens using cement, granite, sand, and metallic waste powder. The findings indicated an enhancement in the linear attenuation coefficient values with elevated metallic waste content. Additionally, S. M. Rasoul Abdar Esfahani et al. (2021)^36^ evaluated concrete mixtures with varying proportions of ground granulated blast furnace slag and copper slag. The results indicated that concrete comprising 60% GGBFS and 100% CS exhibited enhanced radiation shielding properties, rendering it appropriate for structural applications such as healthcare facilities. Additionally, M. A. E. Abdel-Rahman et al. (2019)^37^ conducted research on the attenuation of γ-neutrons and the mechanical characteristics of conventional concrete samples using various additives, including steel and polypropylene fibers, silica fume, and fly ash, with varying cement percentages. The altered concrete mixtures exhibited excellent workability, mechanical strength, and γ-fast neutron impenetrability, beneficial for shielding and protective design. Additionally, M. N. A. Khan et al. (2023)^38^ compared four different percentages of dolerite aggregate in heavy-density concrete (25%, 50%, 75%, and 100%). The results showed that the mix with 75% dolerite aggregate improved gamma ray shielding at all temperatures, increased compressive strength, and decreased mass and density loss. Moreover, several engineered cementitious composite (ECC) formulations for gamma and neutron radiation penetration were evaluated by M. (A) E. M. Ali et al. (2022)^39^. The results demonstrated that the flowability, compressive strength, and tensile strength of ECC were all improved when the amount of nano-silica was increased. Furthermore, ECC attained gamma and neutron attenuation levels of up to 63% and 37%, respectively. M. S. Seyed Mohsen et al. (2024)^40^ aimed to identify the ideal mixture of galena, hematite, and limonite for concrete shielding structures against neutron and gamma radiation. The results indicated that these minerals had a significant gamma absorption capability and enhanced neutron attenuation efficacy. Finally, concretes produced with colemanite material were tested for their photon and fast neutron shielding capabilities by (B) Oto et al. (2019)^41^. The results demonstrated that concretes doped with colemanite were better at shielding fast neutrons than gamma radiation. S.E. Chidiac et al. (2021)^42^ examined the filler effect of commercial boron carbide (B4C) fine powder on the Portland cement hydration reaction and the mechanical and radiation shielding properties of concrete. The results showed that the presence of sassolite in B4C powder retards the hydration reaction for the first 24 h, and significant improvements in concrete strength follow with the increase of boron carbide due to the filler effect. The addition of boron carbide also improved the neutron shielding capabilities of concrete mixes. M. G. El-Samrah et al. (2018)^43^ tested four concrete mixes —goethite-limonite concrete (G.L), barite-limonite concrete (B.L), steel slag-limonite concrete (S.L), and dolomite concrete (D.C) —for their performance as nuclear radiation shielding barriers. Slabs were exposed to fast neutrons and measured for attenuation properties. Results showed G.L. concrete had the highest total removal macroscopic cross-section, but poor attenuation for fast neutrons at 450 °C. B.L. concrete had the highest total linear attenuation coefficients. M. G. El-Samrah et al. (2017)^44^ investigated the performance of four concrete mixes with different coarse aggregates, dolomite, barite, goethite, and steel slag, for radiation shielding applications. The fine aggregates were replaced with 10% silicon fume and 10% fly ash, which affected the samples’ performance. The physical, mechanical, and radiation attenuation properties were studied and compared with those of ordinary concrete. Results showed that goethite-limonite concrete, barite-limonite concrete, steel slag-limonite concrete, and dolomite concrete had good physical and mechanical properties, with higher γ-ray attenuation coefficients. Islam M. Nabil et al. (2024)^45^ evaluated radioactivity levels and associated risks in black sand-separated products from a black sand separation plant in Delta, Egypt. It found that samples of rutile, zircon, and monazite had the highest levels of radioactivity due to their high NORM activity concentrations. The findings suggest that the black sand separation process poses potential risks to human health and the environment, and recommend implementing International Basic Safety Standards and ICRP recommendations to mitigate these risks. Marzieh Hassanpour et al. (2022)^46^ assessed the effects of adding two natural additives—pistachio shells (PS) and date palm leaf (PL)—as partial replacements for conventional sand aggregates on the neutron shielding properties of concrete mixes. Using the Monte Carlo N-Particle code (MCNP), the neutron shielding performance of concrete mixes with varying percentages of PS and PL (1–15%) was investigated. Results showed that higher percentages (10–15%) of these green additives improved neutron shielding effectiveness. Mechanical testing indicated that while the compressive strength of concrete with 1% PS was slightly lower, overall, the incorporation of PS and PL enhanced shielding against fast and thermal neutrons, suggesting they may serve as cost-effective alternatives to enriched boron for neutron shielding applications. Also, Marzieh Hassanpour et al. (2022)^47^ evaluated graphene/h-BN metamaterials as a new material for shielding against neutron radiation using the MCNPX Transport Code. This metamaterial functions effectively as both a thermal and fast neutron moderator and absorber. It comprises hexagonal boron nitride (h-BN) and graphene and was tested against an Am–Be neutron source with an energy range of 100 keV to 15 MeV. The current transmission rate (CTR) was analyzed through Monte Carlo simulations. Results indicated a significant improvement in neutron radiation shielding compared to traditional concrete materials, suggesting that graphene/h-BN metamaterials may serve as a viable alternative to concrete for shielding against neutron radiation.

Although all the aforementioned research work has demonstrated the behavior of concrete shields against various radiations, there remains a lack of information regarding the effects of incorporating goethite, leucoxene, and rutile aggregates on the behavior of concrete. In the present study, these materials were selected based on their distinct physical and chemical properties: goethite for its high iron content and inherent crystalline water; rutile and leucoxene for their combined iron and titanium content, as well as their considerable neutron removal cross-section due to inelastic scattering. Tourmaline was additionally incorporated owing to its boron oxide and iron oxide content. The relatively high density of all the selected aggregates further suggests their potential to enhance both the mechanical and shielding performance of concrete. Therefore, this study aims to examine the appropriateness of utilizing various aggregate combinations, such as goethite, leucoxene, rutile, and tourmaline, as replacements for conventional aggregates to create heavyweight concrete as a shield with acceptable mechanical performance and a probable improved ability to absorb γ-rays and neutrons.

## Materials and experimental program

### Materials

Concrete production relies on cement as a primary component. Thus, Portland cement CEM I (42.5 N) was used in this study following the ASTM C150 standards^48^. The chemical composition of the utilized cement is shown in Table 1. In addition, clean, impurity-free, and regularly tested water from reliable sources was used, in compliance with ASTM C1602/C1602M standards^49^. Additionally, natural siliceous sand, conforming to ASTM C33/C33M standards^50^, was utilized in the production of concrete. Likewise, crushed dolomite aggregate with a maximum nominal size of 20 mm, in accordance with ASTM C33/C33M standards^50^, was used in the manufacture of the normal concrete specimens.

**Table 1:** Chemical composition of utilized cement.

Element	% to Weight
SiO_2_	21.16
Al_2_O_3_	5.01
Fe_2_O_3_	3.76
CaO	64.3
MgO	0.93
SO_3_	2.71
Cl	0.064

**Fig. 1:**
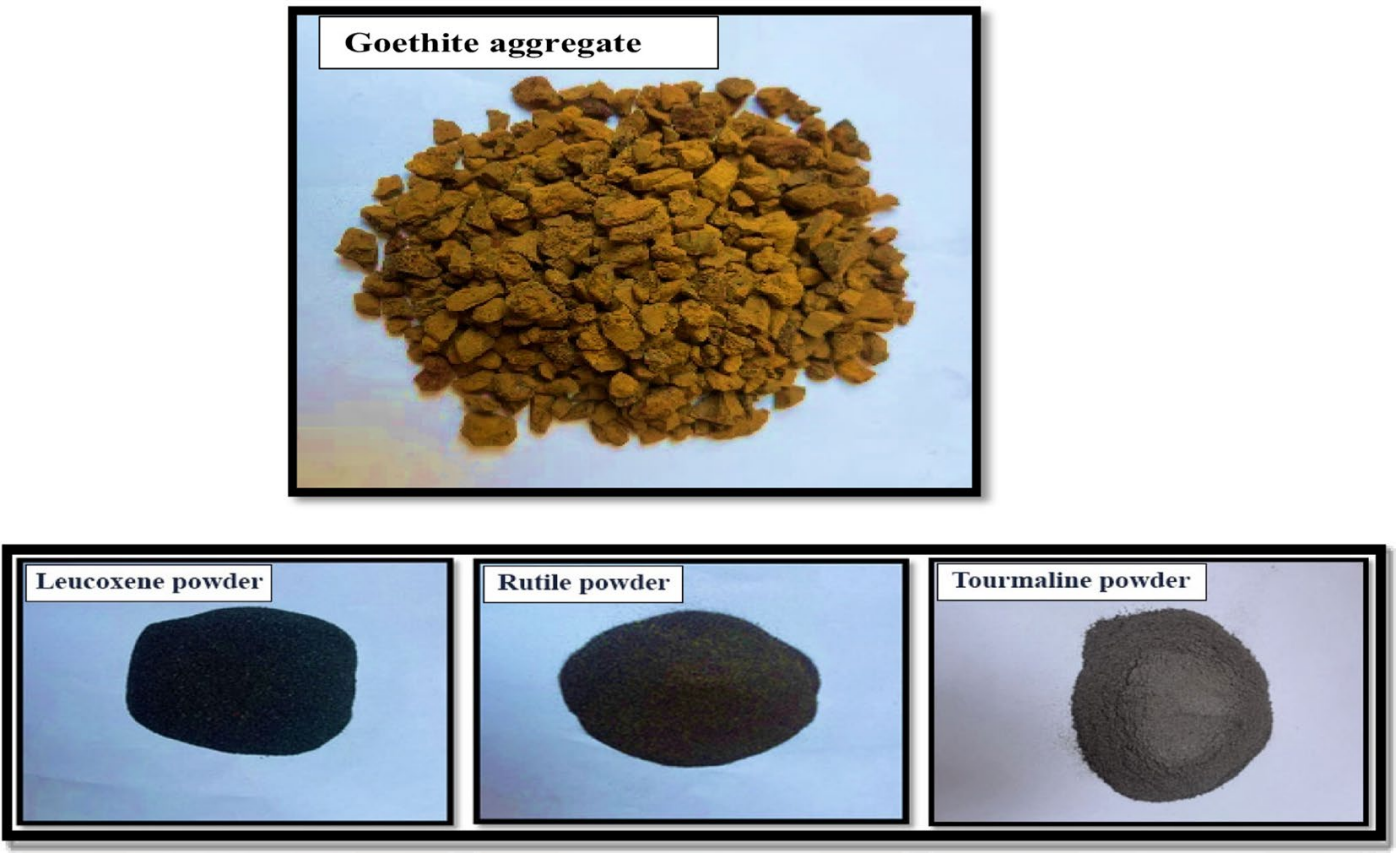
Goethite aggregate, leucoxene, rutile, and tourmaline powders.

On the other hand, a complete replacement for dolomite with goethite aggregate and a partial replacement for silica sand with powdered materials, featuring various types including leucoxene, rutile, and tourmaline, were applied. The chemical composition and physical characteristics of the Goethite aggregate and fine powders are illustrated in Tables 2 and 3. Additionally, Fig. 1 illustrates the various non-traditional aggregates and powders utilized. In addition, a high-range water reducer admixture (HRWRA) was used in accordance with ASTM C494 standards^51^ to enhance the workability and consistency of the concrete mixtures.

**Table 2:** Chemical composition of Goethite, Leucoxene, Rutile, and Tourmaline.

Oxide	% to Weight			
	Goethite	Leucoxene	Rutile	Tourmaline
Al_2_O_3_	0.98	1.5	0	25.14
B_2_O_3_	0	0	0	11.08
CaO	0.27	0	0	0.89
Cl	0	0	0	0
CuO	0	0	0	0
Fe_2_O_3_	80.97	10	0.31	13.02
H_2_O	3.86	0	0	0
K_2_O	0	0	0	1.07
MgO	0.41	0	0	0.51
MnO	0	0	0	0
Na_2_O	0.55	0	0	2.04
P_2_O_5_	3.87	0	0.008	0.59
SiO_2_	8.66	3	0.65	42.65
SO_3_	0.32	0	0.002	0.05
TiO_2_	0.11	85	96.3	0.21
ZrO_2_	0	0	0.95	0
Others	0	0.5	0	0

**Table 3:** Physical characteristics of goethite aggregate and fine powders.

	Goethite	Leucoxene	Rutile	Tourmaline
Odor	Odorless	Odorless	Odorless	Odorless
Form	Coarse	Fine powder	Fine powder	Fine powder
Color	Orange	Black	Black	Black
Density (g/cm^3^)	4.04	4.7	4.23	3.06
Max. nominal size	20 mm	100 μm	100 μm	100 μm

### Concrete mixtures

This experiment resulted in the development of eleven distinct concrete mixtures with different aggregate compositions. A control specimen (NC) with conventional concrete ingredients —cement, high-range water-reducing admixture (HRWRA), silica sand, and dolomite aggregate — was first produced. Then, the (GC) specimen was created in which the coarse dolomite aggregate was replaced entirely by goethite aggregate. Thereafter, the concrete mixtures (GL, GR, and GT) were developed to determine the optimal replacement percentage of silica sand with leucoxene, rutile, and tourmaline powders. The tested replacement percentages were 10%, 20%, and 30% to evaluate their impact on the radiation shielding properties of concrete.

Table 4 provides a detailed representation of the components and their corresponding proportions to cement content in the different mixtures. At the same time, the densities and chemical compositions of the other designed mixtures, both in oxide and elemental forms, are presented in Tables 5 and 6, and 7.

**Table 4:** Concrete mixture proportions.

ID	OPC	w/c	HRWRA	Coarse aggregate		Fine aggregate			
				Dolomite	Goethite	Sand	Leucoxene	Rutile	Tourmaline
NC	1.00	0.50	2%	3.6	0	1.8	0	0	0
GC	1.00	0.50	2%	0	5.57	1.8	0	0	0
GL10	1.00	0.50	2%	0	5.57	1.62	0.32	0	0
GL20	1.00	0.50	2%	0	5.57	1.44	0.63	0	0
GL30	1.00	0.50	2%	0	5.57	1.26	0.94	0	0
GR10	1.00	0.50	2%	0	5.57	1.62	0	0.29	0
GR20	1.00	0.50	2%	0	5.57	1.44	0	0.57	0
GR30	1.00	0.50	2%	0	5.57	1.26	0	0.86	0
GT10	1.00	0.50	2%	0	5.57	1.62	0	0	0.2
GT20	1.00	0.50	2%	0	5.57	1.44	0	0	0.4
GT30	1.00	0.50	2%	0	5.57	1.26	0	0	0.6

Note: all replacement amounts were by weight.

**Table 5:** Density of various concrete mixtures.

ID	NC	GC	GL mixes			GR mixes			GT mixes		
			GL10	GL20	GL30	GR10	GR20	GR30	GT10	1.1.4 GT20	GT30
Density (g/cm^3^)	2.422	3.083	3.131	3.180	3.229	3.120	3.158	3.196	3.093	3.102	3.112

**Table 6:** Chemical compositions in oxide form of designed concrete mixtures.

Oxide	NC	GC	GL mixes			GR mixes			GT mixes		
			GL10	GL20	GL30	GR10	GR20	GR30	GT10	GT20	GT30
H_2_O (%)	7.27	8.08	7.96	7.84	7.73	7.99	7.91	7.81	8.07	8.06	8.04
B_2_O_3_ (%)	0.00	0.00	0.00	0.00	0.00	0.00	0.00	0.00	0.25	0.50	0.75
Na_2_O (%)	0.01	0.35	0.35	0.34	0.34	0.35	0.34	0.34	0.40	0.44	0.49
MgO (%)	8.25	0.36	0.36	0.35	0.35	0.36	0.36	0.35	0.38	0.39	0.40
Al_2_O_3_ (%)	1.54	1.20	1.23	1.26	1.29	1.18	1.17	1.15	1.76	2.32	2.88
SiO_2_ (%)	30.49	28.13	25.80	23.56	21.39	25.81	23.58	21.37	27.02	25.91	24.81
P_2_O_5_ (%)	0.00	2.43	2.40	2.36	2.33	2.41	2.38	2.35	2.44	2.45	2.46
Cl (%)	0.01	0.01	0.01	0.01	0.01	0.01	0.01	0.01	0.01	0.01	0.01
K₂O (%)	0.01	0.00	0.00	0.00	0.00	0.00	0.00	0.00	0.03	0.05	0.08
CaO (%)	51.35	7.44	7.32	7.22	7.12	7.35	7.27	7.19	7.45	7.45	7.46
TiO_2_ (%)	0.00	0.07	3.09	5.94	8.71	3.19	6.13	9.12	0.07	0.08	0.08
MnO (%)	0.01	0.00	0.00	0.00	0.00	0.00	0.00	0.00	0.00	0.00	0.00
Fe_2_O_3_ (%)	0.63	51.41	50.96	50.57	50.20	50.82	50.29	49.72	51.62	51.82	52.03
SO_3_ (%)	0.39	0.51	0.50	0.49	0.49	0.50	0.50	0.49	0.51	0.51	0.51
CuO (%)	0.04	0.00	0.00	0.00	0.00	0.00	0.00	0.00	0.00	0.00	0.00
ZrO_2_ (%)	0.00	0.00	0.00	0.00	0.00	0.03	0.06	0.09	0.00	0.00	0.00
Others (%)	0.00	0.00	0.02	0.03	0.05	0.00	0.00	0.00	0.00	0.00	0.00

**Table 7:** Chemical compositions in elemental form of designed concrete mixtures.

Element	NC	GC	GL mixes			GR mixes			GT mixes		
			GL10	GL20	GL30	GR10	GR20	GR30	GT10	GT20	GT30
H (%)	0.81	0.90	0.89	0.88	0.87	0.89	0.88	0.87	0.90	0.90	0.90
B (%)	0.00	0.00	0.00	0.00	0.00	0.00	0.00	0.00	0.08	0.16	0.23
O (%)	41.92	38.44	38.17	37.90	37.64	38.21	37.99	37.78	38.35	38.26	38.17
Na (%)	0.01	0.26	0.26	0.25	0.25	0.26	0.26	0.25	0.30	0.33	0.36
Mg (%)	4.98	0.22	0.22	0.21	0.21	0.22	0.21	0.21	0.23	0.23	0.24
Al (%)	0.82	0.63	0.65	0.67	0.68	0.63	0.62	0.61	0.93	1.23	1.53
Si (%)	14.25	13.15	12.06	11.01	10.00	12.07	11.02	9.99	12.63	12.11	11.60
P (%)	0.00	1.06	1.05	1.03	1.02	1.05	1.04	1.03	1.07	1.07	1.07
Cl (%)	0.01	0.01	0.01	0.01	0.01	0.01	0.01	0.01	0.01	0.01	0.01
K (%)	0.00	0.00	0.00	0.00	0.00	0.00	0.00	0.00	0.02	0.04	0.06
Ca (%)	36.70	5.32	5.23	5.16	5.09	5.25	5.20	5.14	5.32	5.33	5.33
Ti (%)	0.00	0.04	1.85	3.56	5.22	1.91	3.68	5.46	0.04	0.05	0.05
Mn (%)	0.01	0.00	0.00	0.00	0.00	0.00	0.00	0.00	0.00	0.00	0.00
Fe (%)	0.49	39.96	39.61	39.31	39.02	39.50	39.09	38.65	40.12	40.28	40.44

### Mixture preparation, casting, and curing

**Fig. 2:**
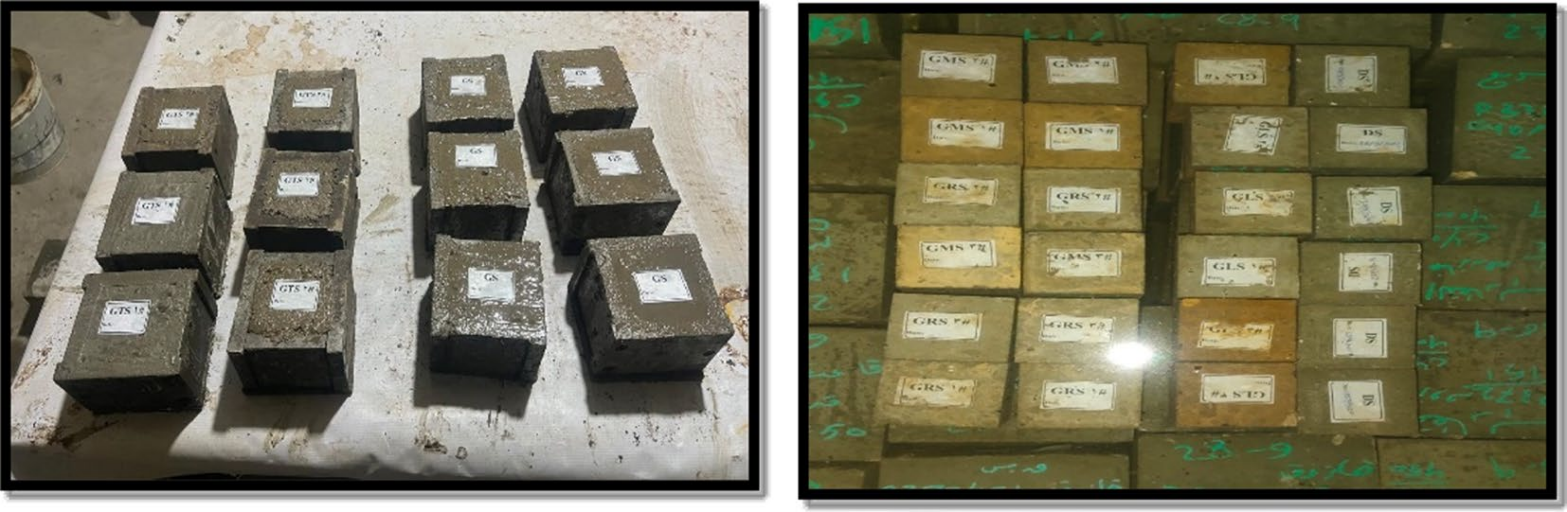
Preparation, casting, and curing of different concrete specimens.

In this investigation, a typical concrete mixing technique was applied. First, the dry components (cement, silica sand, dolomite aggregate, goethite aggregate, leucoxene powder, rutile powder, and tourmaline powder) were blended in the concrete mixer for 2 min to create a uniform dry mixture. In parallel, the HRWRA was mixed with water for the same time. Subsequently, the liquid ingredients were gradually incorporated into the concrete mixer and stirred for 3 min with the dry components, resulting in the development of a wet amalgamation until a consistent and uniform composition was achieved. Thereafter, all the components of concrete were poured into the Molds. The concrete samples were removed from the molds and placed in curing water at a temperature of 20 ± 2 °C and a relative humidity of 95% after 24 h. Then, they were left to cure in natural water until the time of testing. Figure 2 illustrates the various concrete samples during the preparation, casting, and curing processes.

### Experimental program

#### Workability test

Slump cone tests were conducted in this research work to evaluate the effect of incorporating goethite aggregate and leucoxene, rutile, and tourmaline powders in concrete production on the workability of freshly mixed concrete mixtures as per the guidelines of ASTM C143 (Standard Test Method for Slump of Hydraulic-Cement Concrete)^52^.

#### Compressive strength test

The compression test was performed on 100 mm x 100 mm x 100 mm samples at 7 and 28 days of maturity from each concrete mixture, utilizing a compression testing machine produced by ELE with a maximum capacity of 2000 KN. Six identical specimens were assessed from each combination to determine the average measurement for the two testing ages, in accordance with ASTM C39 guidelines^53^. Additionally, the hardness of the dolomite and goethite coarse aggregates was evaluated in this study to assess their relative resistance to crushing under a gradually applied compressive load.

#### Radiation-shielding test

This study examined the effective atomic number (Z_eff_), which assesses shielding against X-rays and γ-rays by characterizing the electronic cloud involved in the photon/atom interaction process. Z_eff_ was computed utilizing the Auto-Zeff software, version 1.7. The software contains a matrix of cross-sections for photon energies ranging from 10 keV to 1 GeV, with atomic numbers (Z) spanning from 1 to 100. The computed cross-sections for the mixture, derived from its constituent weight fractions using linear summation, are compared with the built-in matrix as a function of Z. Subsequently, Z_eff_ is determined via interpolation (b-spline) of the Z values between the neighboring cross-section data at the designated energies. All mixtures incorporating fine powders (rutile, leucoxene, and tourmaline) are expected to demonstrate promising performance in γ-ray shielding due to the presence of high-Z elements that enhance gamma attenuation.

Furthermore, linear attenuation coefficients (µ) were computed for the prepared concrete mixes within the energy range, 10 keV to 10 MeV, using EpiXS software^54^ to assess the shielding capabilities of those mixes against primary energetic X-rays and γ-rays’ photons.

EpiXS is a user-friendly program for Windows^54^ designed for studying photon attenuation, dosimetry, and shielding. The program employs reliable cross-sectional databases such as ENDF/B-VIII’s EPICS2017 and the ENDF/B-VI.8’s EPDL97.

**Fig. 3:**
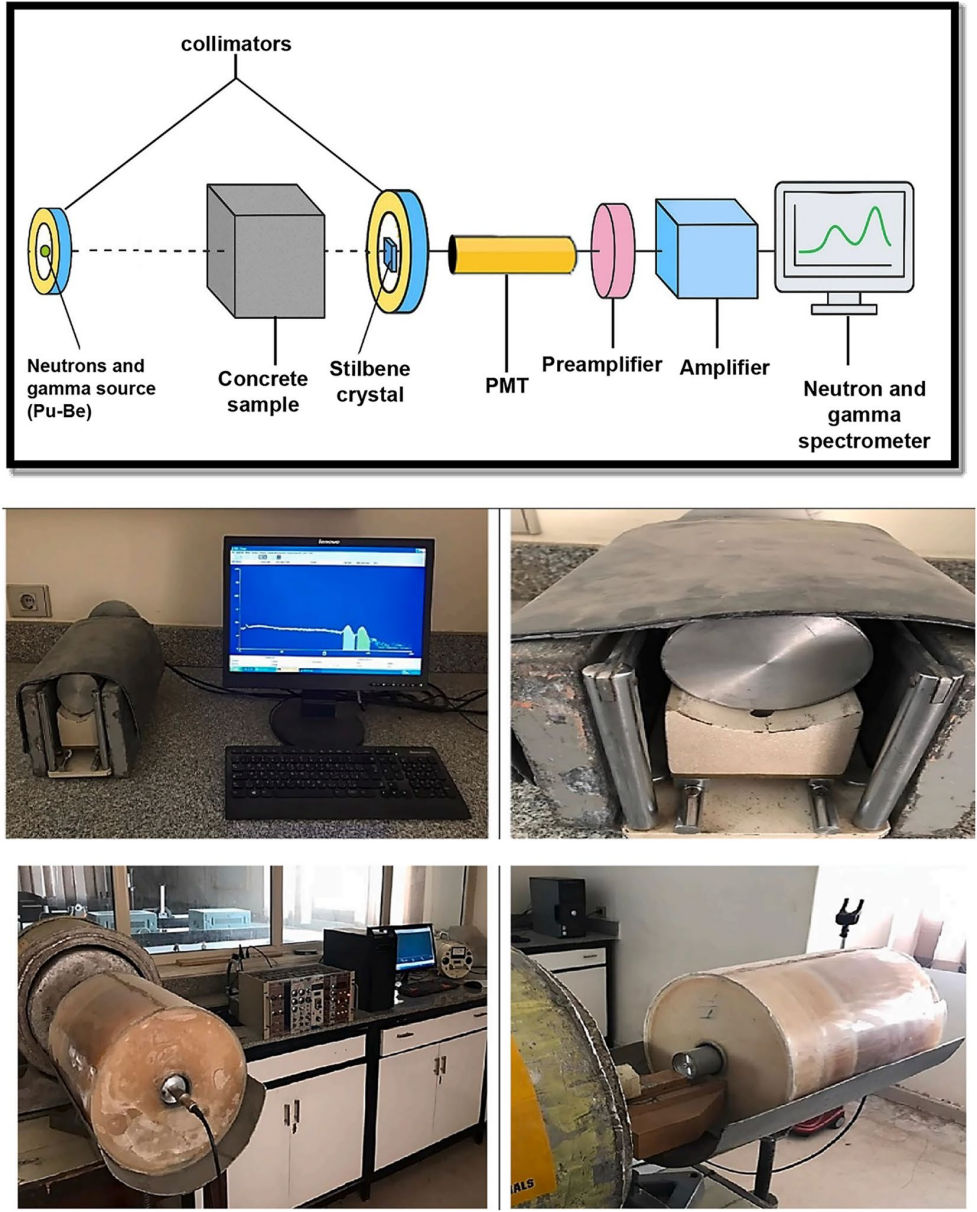
The neutron and gamma spectroscopy system.

A spectroscopy testing technique for fast neutrons and gamma radiation shielding measurements was utilized, as presented in Fig. 3. The system is based on the pulse shape discrimination technique, which effectively differentiates between the integrated counts detected for emitted neutrons and those detected for total gamma rays, based on the shape of the output pulses. The used system was calibrated before the measurements using two neutron radioactive sources: Cf-252 (100 µCi) and Pu-Be (5 Ci) sources, as well as a gamma radioactive source, Cs-137 (100 mCi), which was explicitly used to calibrate the emitted primary gamma rays from the source. However, the measurements performed here in the current study depend on “taking the integral counts only” for both fast neutrons and the total gamma rays without making further spectral analysis, depending on the dependency of the measured counts as a function of the energy. The radioactive source used for the measurements was Plutonium-Beryllium (Pu-Be), primarily a neutron source with a large portion of fast neutrons. Total gamma rays are meant to be the result of the integration between the primary gamma photons emitted by the source itself and the secondary photons produced from any possible radiative capture nuclear reactions between the incident neutrons and the concrete sample being tested.

The emitted radiation is directed toward the concrete sample to study the attenuation capabilities of the proposed mixtures. The transmitted radiation is then detected by a Stilbene scintillation crystal, which is well-known for its excellent pulse shape discrimination (PSD) capabilities, allowing for the effective separation of neutron and gamma events based on their scintillation decay profiles. The light pulses generated in the stilbene crystal are converted into electrical signals by a photomultiplier tube (PMT). These analog signals are first processed through a preamplifier, which conditions and stabilizes the signal, followed by an amplifier, which increases the signal strength to levels suitable for spectral analysis. Finally, the signals are analyzed by a neutron and gamma spectrometer, typically a multichannel analyzer (MCA), which categorizes events based on their energy and pulse shape. This setup enables quantitative and qualitative analysis of radiation fields. It is worth noting that the entire system is horizontally aligned and collimated to ensure that the narrow–beam geometry condition is achieved.

For each of the eleven concrete mixtures, concrete specimens were cast and tested in the form of cubes with thicknesses of 10 mm, 30 mm, 50 mm, and 70 mm. At the same time, the other two dimensions (width and height) were kept constant across all samples to ensure that the exposed cross-sectional area was the same for each specimen. Three cubes were prepared for each thickness/mixture as the measurements were performed on identical triplicates, resulting in 12 cubes per mix design. In total, 132 cubes were cast and tested, providing a comprehensive dataset for evaluating the shielding performance of the proposed concrete mixtures with varying thicknesses.

The fast neutrons removal macroscopic cross section (*∑*_R_) and the linear coefficient of total gamma-ray attenuation (*µ*), as per Beer-Lambert’s law^55–59^, were determined by applying the exponential Eqs. (1 and 2) as follows:

1 $${I_{\left( {\text{x}} \right)}} - {I_{\text{b}}}=({I_{\text{o}}}--{I_{\text{b}}}){{\text{e}}^{( - \sum }}{R^x}^{)}$$

2 $${I_{\left( x \right)}} - {I_{\text{b}}}=({I_{\text{o}}}--{I_{\text{b}}}){{\text{e}}^{( - \mu {\text{ }}x)}}$$

Where *I*_o_ is the initial incident neutrons/photons, *I*_b_ is the recorded background integral counts regarding neutrons/photons, and *I*_x_ denotes the emerging intensity of the neutrons/photons after passing through the thickness (x) of the studied mix. Also, the evaluation of Half Value Layer (HVL), Tenth Value Layer (TVL), and mean free path (MFP) was carried out according to Eqs. (3, 4, and 5), respectively:

3 $${\text{HVL}}\,=\,{\text{ln }}\left( {\text{2}} \right){\text{ }}/\mu$$

4 $${\text{TVL}}\,=\,{\text{ln }}\left( {{\text{1}}0} \right){\text{ }}/\mu$$

5 $${\text{MFP}}\,=\,{\text{1}}/\mu$$

Where the HVL and TVL are the minimum thicknesses required to decrease neutrons / γ-radiation to one-half and one-tenth of their initial intensity, respectively, the MFP is the average distance that neutrons or photons can travel between two successive interactions in the shield.

Finally, to obtain a comprehensive understanding of the neutron shielding effectiveness of the constructed concrete mixes, the macroscopic thermal neutron absorption cross-section of the investigated mixes was calculated at E_n_ = 0.025 eV. JANIS-4.1, an enhanced/modified version of the NEA Java-based Nuclear Data Information System, was used to accomplish this. It provides direct access to evaluated certified nuclear cross-sectional databases, including EXFOR, CENDL, FENDL, JEFF, ENDF, and others^60^.

Thermal neutron macroscopic absorption cross-section Σ_abs_ (cm^− 1^) was calculated based on Eqs. (6–8) as follows:^61,62^

6 $${\left( {{\Sigma _{abs}}/\rho } \right)_i}=\frac{N}{{{\rho _i}}}{\left( {{\sigma _a}} \right)_i}$$

7 $$N=\frac{{{\rho _i}{N_A}}}{{{M_i}}}$$

8 $${\Sigma _{abs}}\left( {{E_n}{\text{~}}={\text{~}}0.025{\text{~}}eV} \right)=\mathop \sum \limits_{1}^{n} {\rho _s}{w_i}{\left( {{\Sigma _{abs}}/\rho } \right)_i}$$

Where; ${\left( {{{\text{\boldsymbol{\upsigma}}}_{\text{a}}}} \right)_{\text{i}}}$, ${{\text{M}}_{\text{i}}}$,${\left( {{{\text{\boldsymbol{\Sigma}}}_{{\text{abs}}}}/{\text{\boldsymbol{\uprho}}}} \right)_{\text{i}}}$, N_A_, and N are the microscopic absorption cross-section for thermal neutrons (cm^2^/atom), the molar mass (g/mol), the macroscopic mass absorption cross-section for thermal neutrons (cm^2^/g), Avogadro’s number (atom/mol), and the atomic density of the element (atom/cm^3^), respectively.

## Results and discussion

### Workability

**Fig. 4:**
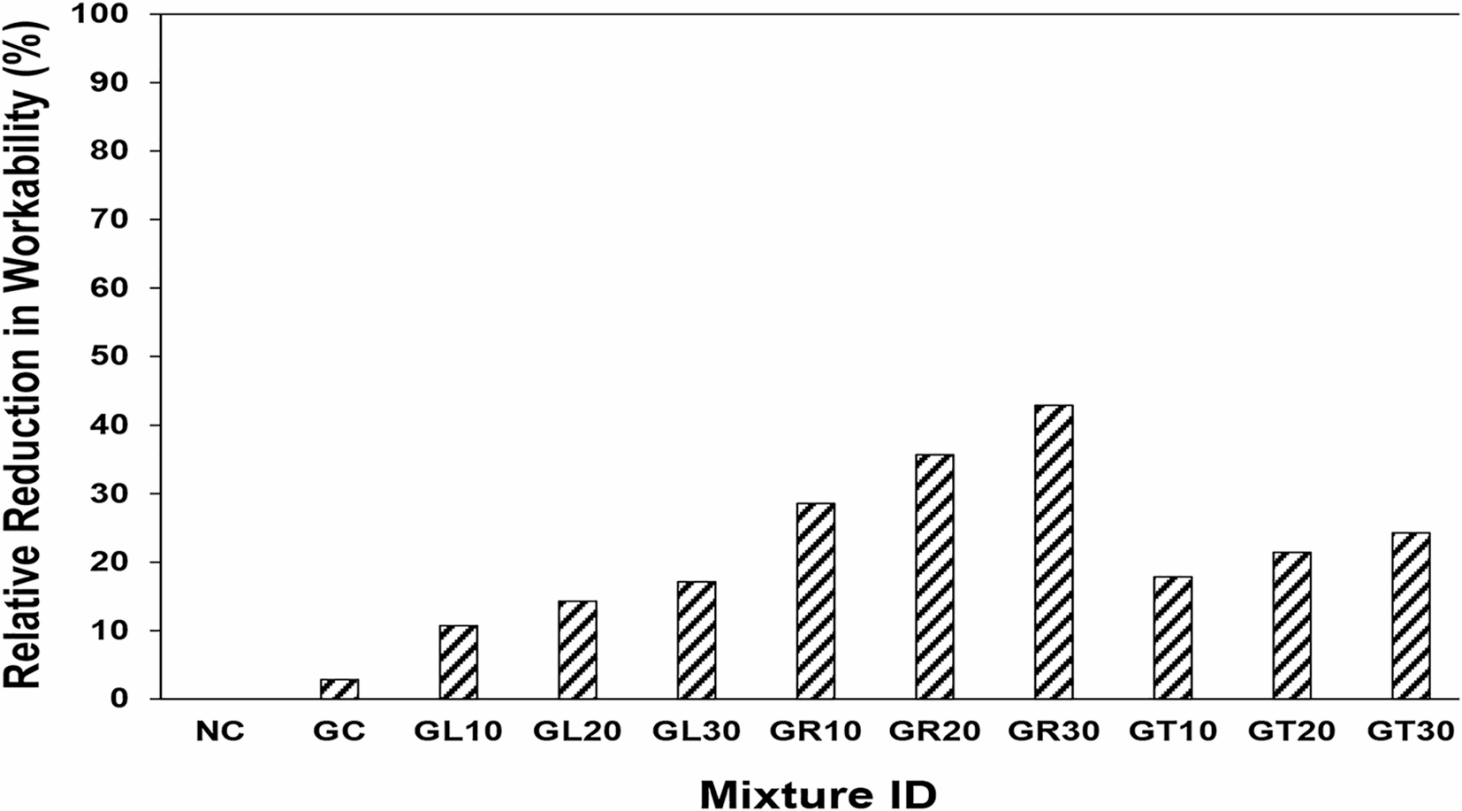
Relative reduction in workability due to different powders’ incorporation.

Figure 4 demonstrates the relative reduction in workability of different concrete mixtures compared to that of the normal concrete (control). Slump cone tests revealed that the conventional concrete mixture could flow under its own weight with a slump drop head of 140 mm. In contrast, the complete replacement of traditional aggregates with goethite aggregate and the partial replacement of silica sand with leucoxene, rutile, and tourmaline powders resulted in an overall reduction in workability. For example, as shown in Fig. 4, the slump drop head of the mixture incorporating goethite aggregate slightly decreased by 2.86% compared to that of the NC control mixture. Similarly, the slump drop head decreased by about 10.71%, 14.29%, and 17.14% due to the incorporation of 10%, 20%, and 30% leucoxene powder, respectively, compared to the NC mixture. Likewise, the slump drop head decreased by approximately 28.57%, 35.71%, and 42.86% due to the incorporation of 10%, 20%, and 30% rutile powder, respectively, compared to the NC mixture. Moreover, the slump drop head decreased by about 17.85%, 21.43% and 24.29% due to 10%, 20% and 30% tourmaline powder’s incorporation, respectively compared to that of NC mixture as shown in Fig. 4. This could be attributed to the addition of fine powders which generally increases the specific surface area of the mixtures, thereby increasing its water demand, which may lead to reduced workability of fresh concrete. It should be noted that the workability reduction can affect the compactness of concrete, acting as an exogenous parameter that can impact its mechanical performance.

### Compressive strength

**Fig. 5:**
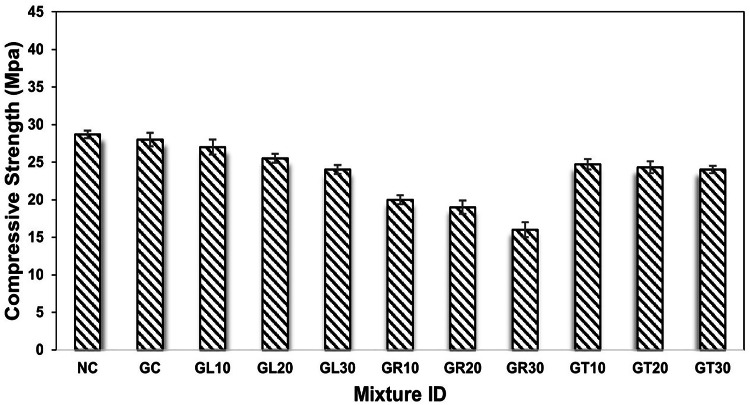
Early age compressive strength of concrete mixtures at 7 days.

**Fig. 6:**
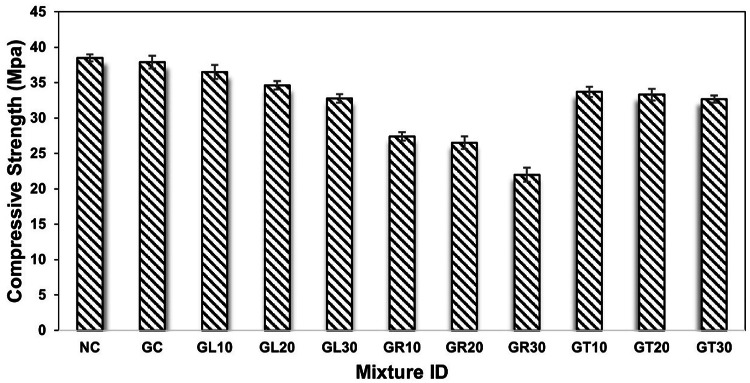
Compressive strength of concrete mixtures at maturity age of 28 days.

The compressive strength test was performed on all concrete samples at 7 and 28 days after curing. Figure 5 illustrates the test results at 7 days, while Fig. 6 shows the compressive strength of the mixtures at 28 days. As presented in the figures, the compressive strength of NC achieved 28.7 MPa and 38.5 MPa at 7 and 28 days of maturity, respectively, while the complete replacement of dolomite with goethite aggregate resulted in a negligible reduction in compressive performance of concrete by about 2.5% and 1.58% compared to NC, respectively. This is attributed to their nearby crushing strength, which achieved a Mohs hardness of 3.6 and 3.55 for dolomite and goethite aggregates, respectively. The results, as mentioned earlier, pave the way for presenting the goethite aggregate as a viable contender for traditional aggregates in concrete production, as its implementation in concrete production still meets the minimum requirements for structural applications according to relevant codes and standards (e.g., ACI 318).

Similarly, the compressive strength of concrete mixtures containing leucoxene, rutile, and tourmaline powder was measured at similar testing ages as shown in the figures. Generally, the compressive strength decreased as the percentage of silica sand replaced by the specified powders increased. For example, compared to normal concrete (NC) at the age of 7 days, the compressive strength was reduced by approximately 3.57%, 9.28%, and 14.29% upon silica sand replacement by 10%, 20%, and 30% with leucoxene powder, respectively. Similarly, the compressive strength of concrete was reduced by approximately 28.57%, 32.14%, and 42.85% due to the partial replacement of silica sand with 10%, 20%, and 30% rutile powder, respectively. Likewise, the decrease was 11.78%, 13.21%, and 14.28% when tourmaline powder partially replaced silica sand by 10%, 20%, and 30%, respectively, as shown in Fig. 5.

On the other hand, the compressive strength test results at 28 days followed similar manner to that obtained at early age strength of 7 days as demonstrated in Fig. 6. For instance, the compressive strength of GL10, GL20, and GL30 specimens was reduced by approximately 3.69%, 8.71%, and 13.46% compared to GC specimen, respectively, due to silica sand replacement by leucoxene powder. Likewise, the compressive strength of GR10, GR20, and GR30 concrete decreased by approximately 27.70%, 30.08%, and 41.95% relative to the GC specimen, respectively, due to the partial replacement of silica sand with rutile powder. In addition, it decreased by 11.08%, 12.14%, and 13.72% in GT10, GT20, and GT30 specimens, respectively, upon replacement of silica sand with tourmaline powder. The observed general reduction in compressive strength of powdered concrete at all testing ages is attributed to the incorporation of weak/fine materials (leucoxene, rutile, and tourmaline powder) in concrete production as a replacement for solid constituents (silica sand). Additionally, the general reduction in workability can increase the porosity of hardened concrete, which consequently leads to a decrease in its compressive strength. Based on the achieved results and following the ACI 318 standard, incorporating leucoxene or tourmaline powders by up to 30% as a replacement for silica sand still achieved the minimum requirements of concrete’s compressive strength for structural applications. In comparison, it is not preferable to incorporate the rutile powder in concrete production, as it leads to a general weakness in the concrete’s compressive strength.

### Radiation shielding performance

#### Computed effective atomic number and linear attenuation coefficient

**Fig. 7:**
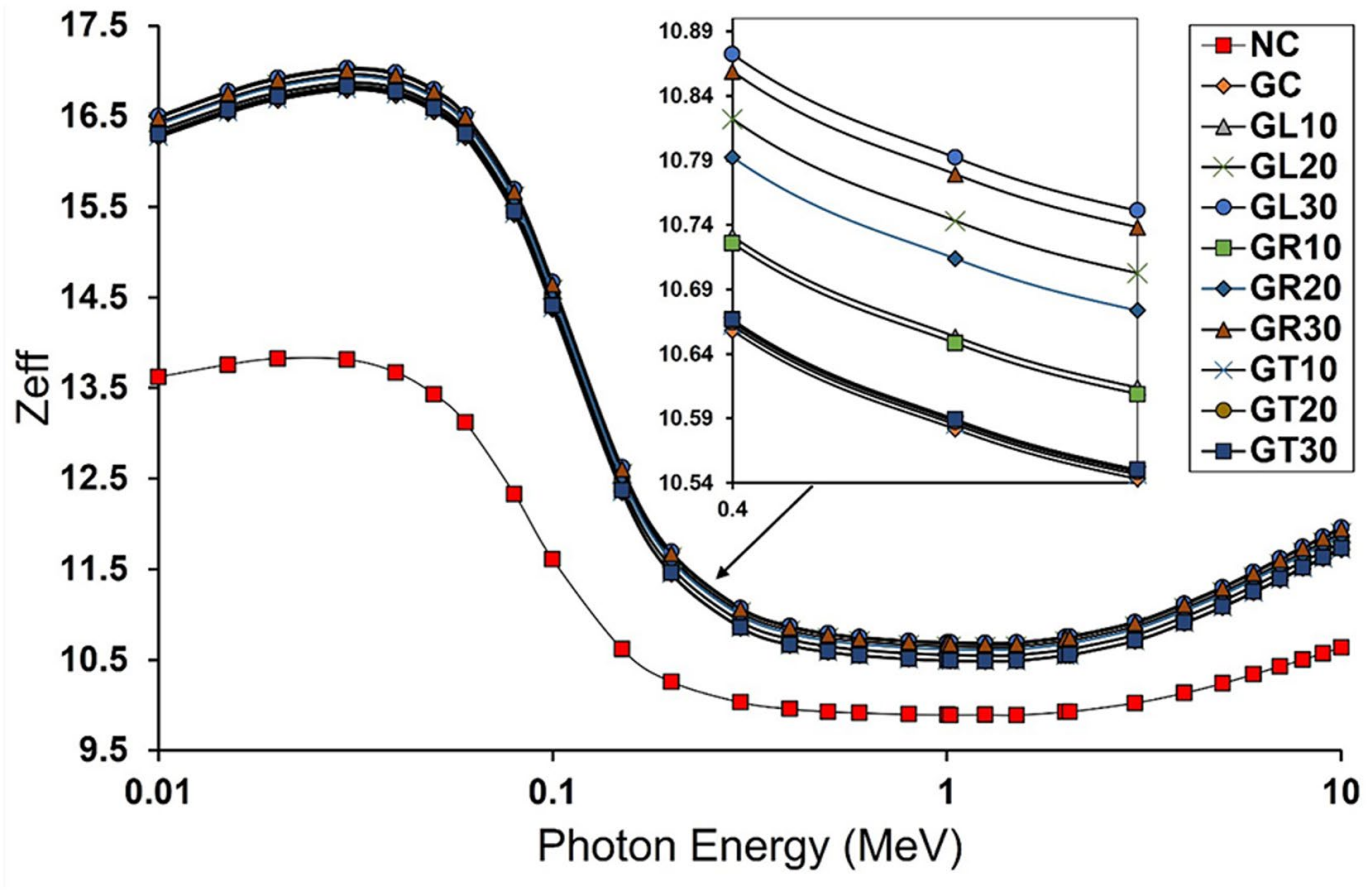
Z_eff_ values for the studied concrete mixes as a function of the photon energy.

Based on Fig. 7, it can be observed that all modified concrete mixes possess effective atomic numbers for the entire studied energy range, which are greater than those for the NC mix, which is considered the traditional control mix in this study. The former does make sense, as all modified mixes contain goethite as a coarse aggregate, besides replacing the traditional fine aggregate (sand) with leucoxene, rutile, or tourmaline, knowing that all these aggregates comprise appreciable amounts of high-Z elements such as Fe and Ti. Moreover, even if the differences between the modified concrete mixes are not significant, GL30 and GR30 still exhibit the highest Z_eff_ values among the studied mixes, which can be attributed to the high contents of Fe and Ti, high-Z elements.

Notably, the Z_eff_ values were found to be highest in the low energy range, as the photoelectric mechanism, which significantly depends on the atomic numbers of the shield constituents (Z^4.5^), is the dominant mechanism. The lowest Z_eff_ values can be observed at the intermediate energies due to the dominancy of the Compton scattering mechanism which is the lowest dependent mechanism on the atomic number then, the Z_eff_ values regain its increasing trend, but not with same manner like the case with the lowest energies, at the high energy range due to the significant contribution of the pair production mechanism which correlates with the squared value of the atomic number (Z^2^).

**Fig. 8:**
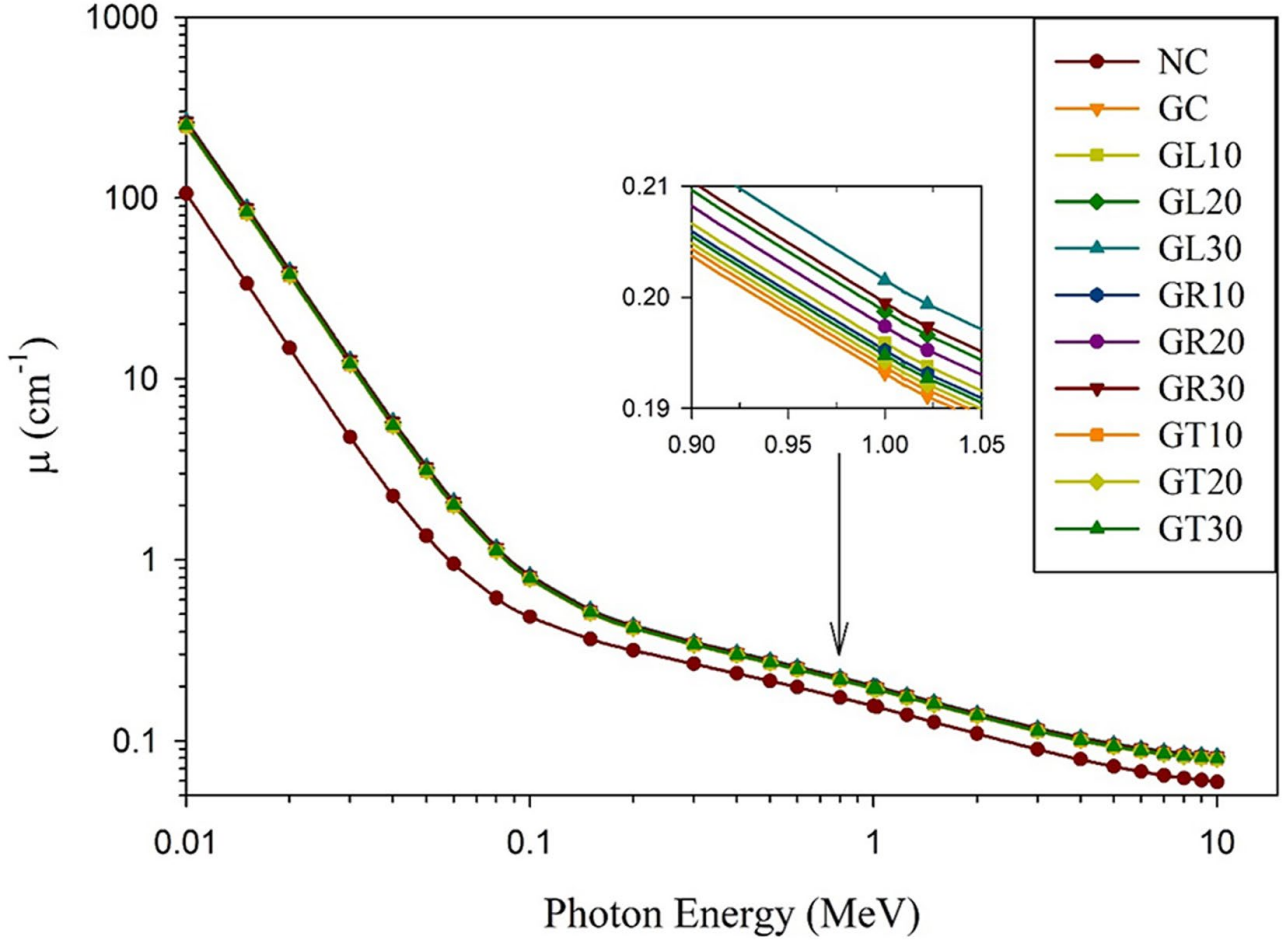
Computed linear attenuation coefficients for the studied concrete mixes.

Since the effective atomic number is determined by the primary modes of gamma ray interaction with matter, just as the linear attenuation coefficient is, the calculated linear attenuation coefficients for the investigated mixes were found to be consistent with the obtained Z_eff_ values, as shown in Fig. 8. Even though there are minor variations across the goethite-based concrete mixes, they are still superior in terms of γ-ray shielding when compared to the NC mix, which is the conventional concrete mix with GL30 and GR30 placed on top of the mixes under investigation.

The reason for this is that the highest high-Z elements content, represented by the combined iron (Fe) and titanium (Ti) content, is found in GL30 and GR30 mixes (about 44%), which also have the highest densities among the studied mixes: 3.229 and 3.196 g/cm^3^, respectively.

#### Results of fast neutrons attenuation measurements

**Fig. 9:**
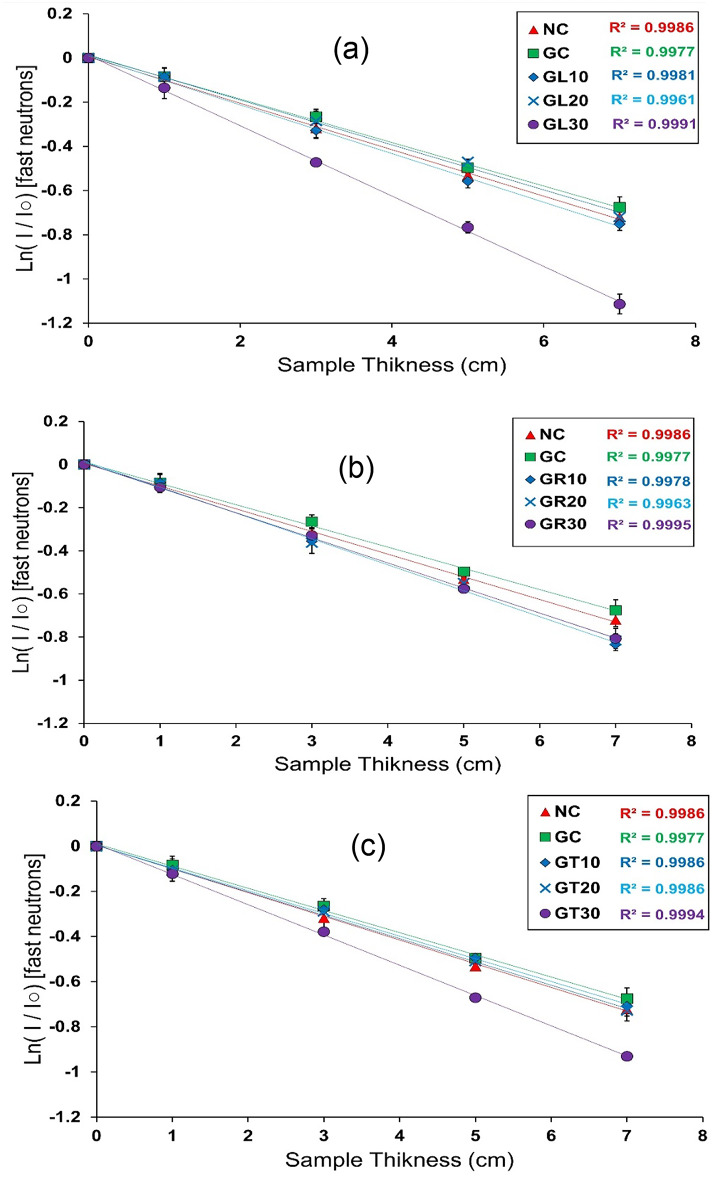
Transmission of fast neutrons attenuation measurements for NC, GC, (a) GL, (b) GR, and (c) GT specimens.

The concrete mixes used in this study were tested to determine the best mix among them, considering fast neutrons attenuation. The transmission curves obtained for fast neutrons attenuation measurements are shown in Fig. 9a–c.

Using the compiled transmission curves, the slope for each curve was taken to represent the fast neutrons removal macroscopic cross section (Σ_R_).

**Fig. 10:**
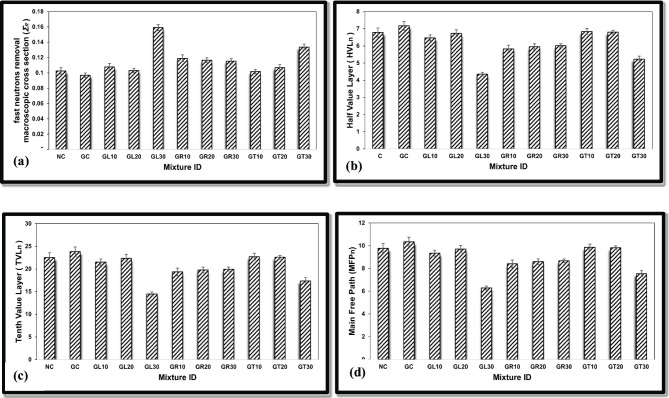
Fast neutrons parameters for different concrete mixtures; (a) Σ_R_, (b) HVL_n_, (c) TVL_n_, and (d) MFP_n_.

Based on the experimentally obtained (Σ_R_), the required shielding parameters, such as HVL_n_, TVL_n_, and MFP_n_, were calculated using Eqs. (3–5) to fully assess the concrete mixes under study, considering their capabilities in shielding against fast neutrons, as presented in Fig. 10a–d.

Based on the obtained results, the GL30 mix then GT30 mix were found to be the best, considering the removal of fast neutrons among the studied mixes.

Considering the light elements’ content, we can observe that both GL20 and GR30 samples possess, roughly, the same percentages, nearly 38.5%. Furthermore, both of them include almost the same percentage of Fe & Ti, which equals about 44%, as shown above while discussing the chemical analyses of the prepared mixes. Moreover, this percentage of Fe & Ti is the most significant among the other concrete samples under study, which adds credibility to both samples considering fast neutron removal relying on the inelastic interaction mechanism, especially since iron and titanium have relatively notable fast neutron mass removal cross-sections compared to other intermediate and heavy elements.

Speaking about the density as a determining factor while evaluating fast neutrons attenuation, knowing that GL30 possesses the highest density among all tested samples (ρ_GL30_= 3.229 g/cm^3^), then GR30 comes in the second place (ρ_GR30_ = 3.196 g/cm^3^), a logical interpretation could rely on the linear summation rule according to Eq. (9) as follows:

9 $$\mathop \sum \limits_{R} =\mathop \sum \limits_{i} {\rho _s}{w_i}{\left( {\frac{{{\sum _R}}}{\rho }} \right)_i}$$

where; ${\rho _s}$ Is the density of the shield, ${w_i}$ Is the weight fraction of the ith element, ${\left( {\frac{{{\sum _R}}}{\rho }} \right)_i}$is the mass removal cross-section is that of the ith element.

According to the former equation, we can thus observe a direct relationship between the shield’s density and macroscopic removal cross-section, since a higher density, which in turn results in a higher nucleic density, implies a greater probability of interaction between the incident fast neutron and the shield’s constituents, particularly through scattering mechanisms.

The reason the light elements’ content, especially the hydrogen content, in all prepared concrete mixes is very close is that the prepared concrete mixes, having been poured into molds, were covered with thin polyvinyl sheets to prevent the evaporation of retained water used during the mixing process. Subsequently, the demolded mixtures were promptly transferred to the wet curing phase, where all samples were submerged underwater for 28 days to prevent any loss of water during the hardening process.

Upon the completion of the curing stage, all tests were conducted concurrently, thereby preserving the confined water, whether that used during the mixing process or the crystalline water associated with goethite.

The corresponding shielding thicknesses, as HVL_n_, TVL_n_, and MFP_n_, confirm the observations mentioned above, as both concrete mixes, GL30 and GT30, show the lowest values for these thicknesses among the studied mixes.

#### Results of total gamma-rays attenuation measurements

**Fig. 11:**
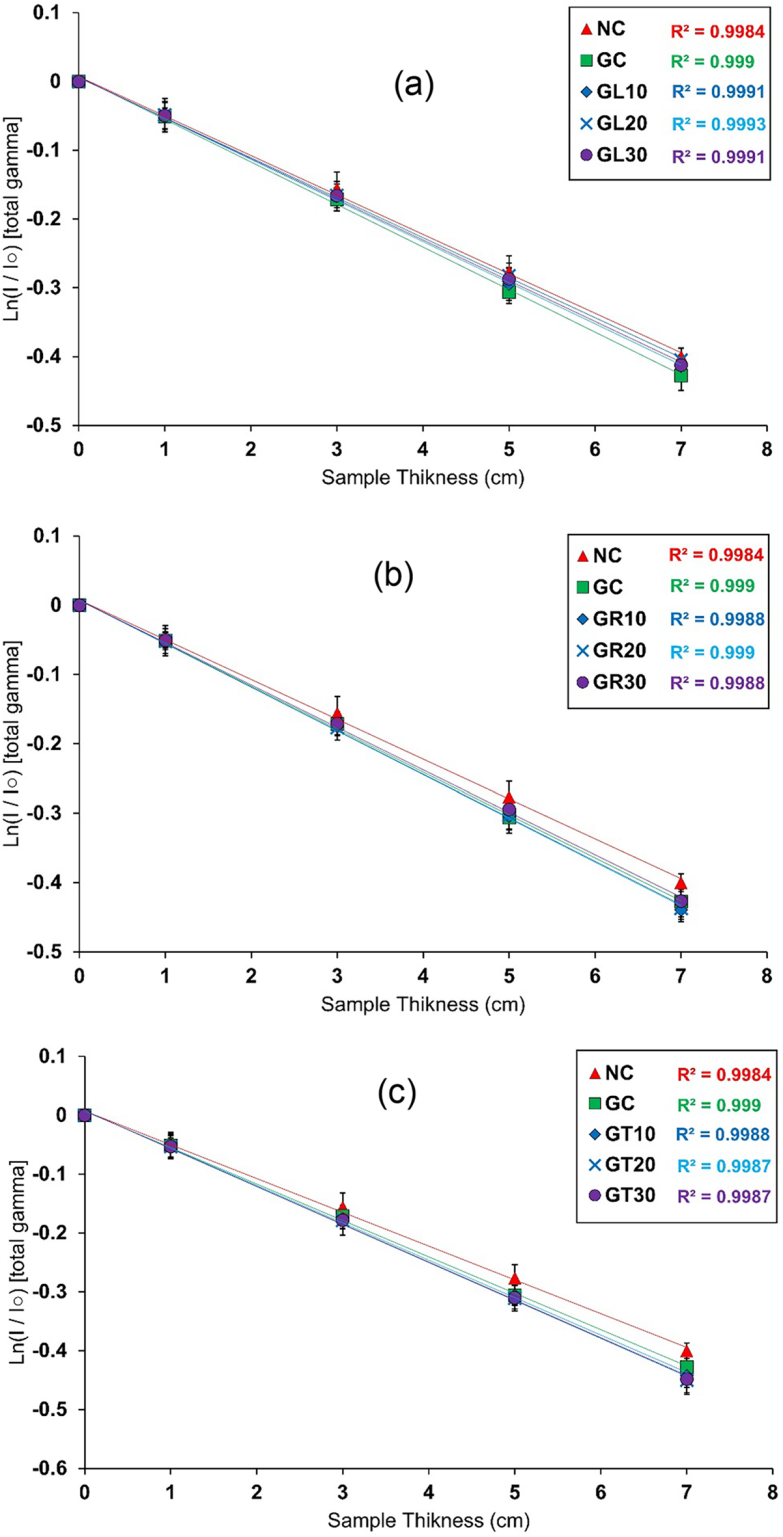
Transmission of gamma-rays attenuation measurements for NC, GC, (a) GL, (b) GR, and (c) GT specimens.

To determine the optimal mix for gamma-ray attenuation, the concrete mixes used in this study underwent testing. The transmission curves acquired for gamma-ray attenuation measurements are illustrated in Fig. 11a–c.

The resulting transmission curves were used to determine the slope for each curve, which represents the linear attenuation coefficient for total gamma (µ).

**Fig. 12:**
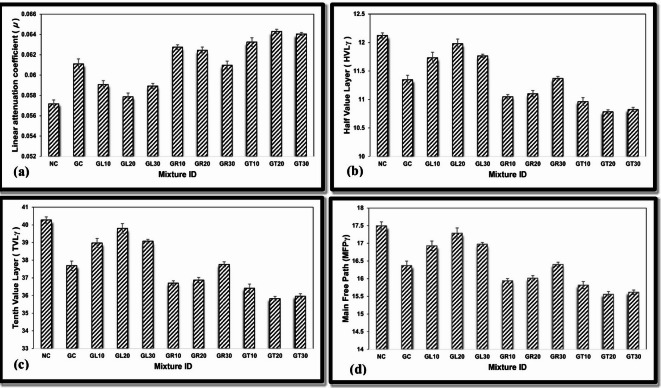
Gamma-ray parameters for different concrete mixtures; (a) µ, (b) HVL_γ_, (c) TVL_γ_, and (d) MFP_γ_.

Using the experimentally derived (µ), the necessary shielding parameters, including HVLγ, TVLγ, and MFPγ, were computed according to Eqs. (3–5) to comprehensively evaluate the concrete mixtures under investigation for their efficacy in shielding against total gamma radiation, as shown in Fig. 12a–d.

As was noted before, total gamma-rays are the combination of the primary gamma-rays emitted from the source and the secondary ones emitted from any possible nuclear interactions between the incident neutrons and the shielding material.

Based on the former study, GT concrete mixes, specifically GT20 and GT30, were found to have the most significant linear attenuation coefficient among the studied mixes.

The main reason is that the existence of boron in these mixes competes with hydrogen and the other light elements, considering absorbing the thermalized neutrons, which leads to a notable decrease in secondary gamma-rays, knowing that the ^1^H (n,γ) ^2^H reaction emits γ-rays with energy equal to 2.2 MeV with an absorption cross section equal to about 0.332 barns. However, when boron absorbs thermalized neutrons, ^10^B (n,α) ^7^Li, the reaction may produce lower energetic secondary gamma-rays with an energy equal to 0.48 MeV only. Knowing the absorption cross section for the former reaction with ^10^B is 3840 barns, and approximately 768 barns in the current case, considering that the incorporated boron is natural boron with only 20% of ^10^B.

In addition to the former, both GT20 and GT30 mixes have high densities among the studied mixes, equal to 3.102 g/cm³ and 3.0112 g/cm³, respectively.

The corresponding shielding thicknesses, as HVL_γ_, TVL_γ_, and MFP_γ_, confirm the observations mentioned above, as both concrete mixes, GT20 and GT30, show the lowest values for these thicknesses among the studied mixes.

According to the acquired results of static and shielding testing, and following the ACI 318 and 349 standards, incorporating leucoxene or tourmaline powders as a replacement for silica sand in concrete production by up to 30% could achieve the minimum requirements of structural and shielding applications. While incorporating rutile powder into a cementitious paste could be a better application for its shielding effectiveness, it would not lead to a general weakness in the concrete’s compressive strength.

#### Thermal neutrons absorption assessment

**Fig. 13:**
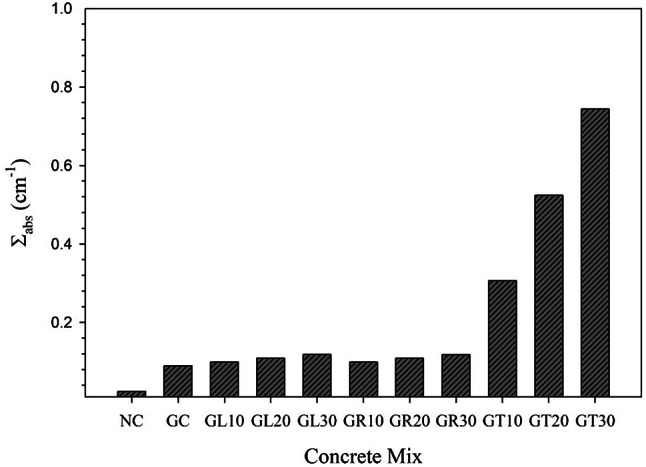
Macroscopic thermal neutrons absorption cross-sections (Σ_abs_) for the studied concrete mixes.

Regarding the ability of the concrete mixes under investigation to absorb thermalized neutrons, the comprehensive computations, computed macroscopic absorption cross-section (Σ_abs_) at E_n_ = 0.025 eV, and the associated necessary shielding thicknesses, HVL_thn_, TVL_thn_, and λ_thn_, are clarified through Fig. 13; Table 8.

**Table 8:** Calculations of thermal neutrons macroscopic absorption cross-sections for the studied concrete mixes.

Ele.	Σ_abs_/ρ (cm^2^/g)	Σ_abs_ (cm^-1^)										
		NC	GC	GL10	GL20	GL30	GR10	GR20	GR30	GT10	GT20	GT30
H	2.00E-01	8.18E-03	1.16E-02	1.16E-02	1.17E-02	1.17E-02	1.16E-02	1.16E-02	1.16E-02	1.16E-02	1.16E-02	1.17E-02
B	4.20E + 01	0.00E + 00	0.00E + 00	0.00E + 00	0.00E + 00	0.00E + 00	0.00E + 00	0.00E + 00	0.00E + 00	2.16E-01	4.34E-01	6.52E-01
O	6.39E-06	1.35E-05	1.58E-05	1.59E-05	1.60E-05	1.62E-05	1.59E-05	1.60E-05	1.61E-05	1.58E-05	1.58E-05	1.58E-05
Na	1.38E-02	6.97E-06	2.31E-04	2.34E-04	2.29E-04	2.32E-04	2.33E-04	2.36E-04	2.30E-04	2.67E-04	2.95E-04	3.22E-04
Mg	1.56E-03	3.92E-04	2.21E-05	2.24E-05	2.17E-05	2.21E-05	2.23E-05	2.16E-05	2.18E-05	2.31E-05	2.32E-05	2.43E-05
Al	5.21E-03	2.15E-04	2.10E-04	2.21E-04	2.31E-04	2.38E-04	2.13E-04	2.12E-04	2.11E-04	3.12E-04	4.13E-04	5.16E-04
Si	3.54E-03	2.54E-03	2.99E-03	2.78E-03	2.58E-03	2.38E-03	2.77E-03	2.56E-03	2.35E-03	2.88E-03	2.77E-03	2.66E-03
P	3.29E-03	0.00E + 00	2.24E-04	2.25E-04	2.24E-04	2.25E-04	2.24E-04	2.25E-04	2.25E-04	2.27E-04	2.27E-04	2.28E-04
Cl	5.71E-01	2.87E-04	3.66E-04	3.72E-04	3.77E-04	3.83E-04	3.70E-04	3.75E-04	3.79E-04	3.67E-04	3.68E-04	3.69E-04
K	3.22E-02	0.00E + 00	0.00E + 00	0.00E + 00	0.00E + 00	0.00E + 00	0.00E + 00	0.00E + 00	0.00E + 00	4.14E-05	8.30E-05	1.25E-04
Ca	6.61E-03	1.22E-02	2.26E-03	2.25E-03	2.26E-03	2.26E-03	2.25E-03	2.26E-03	2.26E-03	2.26E-03	2.27E-03	2.28E-03
Ti	8.06E-02	0.00E + 00	2.07E-04	9.71E-03	1.90E-02	2.83E-02	9.99E-03	1.95E-02	2.93E-02	2.07E-04	2.60E-04	2.61E-04
Mn	1.45E-01	7.33E-05	0.00E + 00	0.00E + 00	0.00E + 00	0.00E + 00	0.00E + 00	0.00E + 00	0.00E + 00	0.00E + 00	0.00E + 00	0.00E + 00
Fe	2.78E-02	6.86E-04	7.12E-02	7.17E-02	7.22E-02	7.28E-02	7.12E-02	7.13E-02	7.14E-02	7.17E-02	7.22E-02	7.27E-02
Tot. Σ_abs_ (cm^-1^)		0.0246	0.0893	0.0991	0.1088	0.1185	0.0989	0.1083	0.1179	0.3061	0.5241	0.7437
HVL_thn_ (cm)		28.151	7.7655	6.9944	6.3709	5.8486	7.0106	6.4006	5.8789	2.2647	1.3225	0.9321
TVL_thn_ (cm)		93.515	25.796	23.234	21.163	19.428	23.288	21.262	19.529	7.5233	4.3931	3.0963
λ_thn_ (cm)		40.613	11.203	10.091	9.1913	8.4377	10.114	9.2341	8.4814	3.2673	1.9079	1.3447

Considering the thermalized neutrons absorption capabilities for the studied mixes, it has been noticed that all prepared goethite-based concrete mixes showed significant superiority above the traditional NC mix. However, exceptional superiority was detected with the GT mixes that contain fine tourmaline replacement with silica sand. For instance, this superiority reaches a 2923% increase compared to the NC mix.

The boron oxide (B_2_O_3_) content exists in the utilized tourmaline powder that is added in the prepared GT mixes was the main reason for the above mentioned superior thermal neutron absorption capability as elemental boron even in its natural form (about 80% Boron-11 plus 20% Boron-10) still considered as one of the most known neutron absorbers that has notable microscopic absorption cross-section which equals 765 barn. Boron absorbs thermalized neutrons through the nuclear absorption reaction; ^10^B (n,α) ^7^Li, through which the incident neutron is completely captured and ends its trip through the shielding medium.

Based on the former, the required shielding thicknesses to fully absorb the thermalized neutrons are significantly reduced when using GT mixes compared to the other studied mixes, especially the traditional NC mix.

## Conclusions

In this investigation, a synergetic static and radiation-shielding behavior of non-traditional concretes were presented. The conclusions were drawn as follows:

- Goethite aggregate proved its worth to act as a contender to conventional dolomite in concrete. Its implementation in concrete production led to a negligible reduction in the workability by about 2.86% and compressive strength by about 1.58% compared to normal concrete.
- Partial replacement of silica sand with leucoxene/rutile/tourmaline powders generally reduced the workability of concrete due to the increased surface area of powders. Also, the concretes’ compressive strength was reduced by up to 1.29%, 42.85%, 14.28% at 7 days and 13.46%, 41.95%, 13.72% at 28 days of maturity, respectively.
- Among all investigated concrete mixes, GL30 and GT30 exhibited the highest efficiency in attenuating fast neutrons, which is attributed to their higher densities and elevated Fe and Ti contents that enhance inelastic scattering interactions.
- GT20 and GT30 concrete mixes exhibited the most effective total (primary + secondary) γ-ray attenuation among all studied compositions. Their superior performance is primarily attributed to the presence of boron in tourmaline, which efficiently absorbs thermal neutrons and consequently reduces the emission of high-energy secondary γ-rays.
- The goethite-based concrete mixes demonstrated a significant enhancement in thermal neutron absorption compared to normal concrete, with GT mixes containing fine tourmaline achieving up to a 2923% increase in absorption efficiency.
- The superior performance of GT mixes is primarily attributed to the boron oxide (B₂O₃) present in tourmaline, which enables effective neutron capture through the ^10^B(n,α)^7^Li reaction, thereby reducing the required shielding thickness for full thermal neutron absorption.
- According to the acquired results of static and shielding testing, incorporating leucoxene or tourmaline powders as a replacement for silica sand in concrete production by up to 30% could achieve the minimum requirements of structural and shielding applications. Implementing rutile powder in cementitious paste could be a better application due to its shielding effectiveness.
- This research paves the way for the feasibility of conducting more investigations on non-traditional concretes with enhanced radiation-shielding characteristics in critical infrastructures.

## Data Availability

All data generated or analyzed during this study are included in this published article.
